# The effectiveness of physical activity interventions in improving higher education students’ mental health: A systematic review

**DOI:** 10.1093/heapro/daae027

**Published:** 2024-04-02

**Authors:** Samantha Donnelly, Kay Penny, Mary Kynn

**Affiliations:** The University of the West of Scotland, Division of Sport, Exercise and Health, Stephenson Place, Hamilton International Technology Park, South Lanarkshire G72 0LH, UK; University of Aberdeen, School of Medicine, Medical Sciences and Nutrition, Foresterhill, Aberdeen AB25 2ZD, UK; Curtin University, Faculty of Science and Engineering, Wark Avenue, Bentley, WA 6102, Australia

**Keywords:** university students, college students, physical activity, exercise, interventions, mental health

## Abstract

Traditional interventions aiming to improve students’ mental health and quality of life include meditation or canine therapy. The development of physical activity-related interventions has increased over the past decade. We aimed to review all studies using physical activity for improving the mental health and quality of life in higher education students whilst describing the interventions, measurements and effectiveness. A systematic search of six electronic databases including: ProQuest, MEDLINE, Embase, CINAHL, SPORTDiscus and CENTRAL, was conducted following PRISMA guidelines. Randomized or non-randomized controlled trial physical activity-related interventions involving higher education students aiming to improve their mental health and quality of life were included. Searches yielded 58 articles with interventions involving martial arts, sport, mind–body exercises and anaerobic exercises. Psychological measures varied across studies including the State Trait Anxiety Inventory, Beck Depression Inventory and the Perceived Stress Scale. Over half of the studies included in this review (*n* = 36) were effective in improving students’ mental health or quality of life. Findings from our review suggest that interventions aiming to be effective in improving students’ mental health quality of life should aim to deliver moderate-vigorous intensity exercises such as dance or Pilates. This systematic review was based on a published protocol in PROSPERO (registration number: CRD42022325975).

**Contribution to Health Promotion**

- We searched academic papers to determine the effectiveness of physical activity for improving the mental health and quality of life of higher education students.
- We identified various physical activity-related interventions which are effective in improving students’ mental health and quality of life.
- We offer recommendations for the design of future interventions aiming to improve the mental health and quality of life of higher education students through physical activity.

## BACKGROUND

The benefits associated with being physically active are well-recognized. Regular participation in physical activity (PA) has been associated with positive long-term influences on obesity, cardiovascular heart disease and type 2 diabetes mellitus in adults (Reiner *et al*., 2013) and is also associated with lower depressive symptoms, anxiety and stress (UK Government, 2019). Interest in the role of PA on mental health and quality of life (QoL) has increased over the past few years due to the Coronavirus Disease-19 (COVID-19) outbreak (Ozdemir *et al*., 2020). Nevertheless, the known improvements in outcomes relating to mental health (e.g. depression, anxiety, stress, etc.) and QoL (e.g. personal health (physical, mental and spiritual), relationships, education status, work environment, social status, wealth, etc.) through PA have been observed for decades (Saxena *et al*., 2005; Acree *et al*., 2006; Bize *et al*., 2007; Farris and Abrantes, 2020). To gain the health benefits associated with being physically active, the World Health Organization (WHO) provide PA guidelines for adults aged 18–64 years (World Health Organization, n.d.). These outline that adults should participate in at least 150–300 min of moderate-intensity aerobic PA or at least 75–150 min of vigorous-intensity activity weekly. The WHO also recommends that adults should also participate in muscle-strengthening activities and limit sedentary time.

Higher education (HE) students are largely recognized as inactive (Pengpid *et al*., 2015), with a decline observed in activity from high school into HE (Bray and Born, 2004). College students indicate that the quality of on-campus facilities, need for social support to encourage exercise and lack of time and motivation are barriers to PA (LaCaille *et al*., 2011).

In HE students, meeting PA guidelines has been associated with improved physical health and mental health (Murphy *et al*., 2018), and less academic stress compared to students who are inactive (Gasiūnienė and Miežienė, 2021). It is unsurprising that a recent systematic review noted a positive relationship between PA and academic performance in HE students across the USA, France and China (Wunsch *et al*., 2021). Alarmingly, findings from a study in 23 low-, middle- and high-income countries found that 41.4% of university students were physically inactive (Pengpid *et al*., 2015) ranging from 21.9% in Kyrgyzstan to 80.6% in Pakistan. Whilst the benefits of PA for HE students are clear in relation to health and academic performance, globally these students are inactive.

HE is a time of increased autonomy and self-development for students, however, poor diet and PA behaviours can develop, with research highlighting failings of HE organizations in the promotion of PA for their students (Keating *et al*., 2005). Additionally, many HE students face issues with their mental health and QoL ranging from anxiety to alcohol use disorders (Castillo and Schwartz, 2013). HE institutions are promising settings to promote PA opportunities, as the target population can be easily reached, whereby interventions to improve the mental health of HE students can be easily administered (Martinez *et al*., 2016). In turn, the improvement of mental health through participation in PA can have positive effects on academic performance (Al-Drees *et al*., 2016). To attempt to enhance health-related behaviours in HE students, previous studies have relied on psychological-based therapies to remedy HE student’s mental health problems (Reiner *et al*., 2013; Binfet, 2017). Findings from a systematic review and meta-analysis exploring the use of cognitive behavioural therapies including mindfulness programmes for the treatment of stress, depression and anxiety in students found a medium effect size for stress and anxiety, but a small effect for depression (González-Valero *et al*., 2019). Studies examining the effects of canine therapy on university students’ stress have shown that spending time with therapy canines significantly reduces stress (Binfet, 2017; Binfet *et al*., 2018). Over the past decade, there has been an increase in interventions employing PA or exercise to improve HE student’s mental health and QoL. Although a review has been conducted examining the effectiveness of interventions targeting PA, nutrition and healthy weight for HE students (Plotnikoff *et al*., 2015), this current review is the first to investigate the effectiveness of PA-related interventions in improving the QoL and mental health outcomes in HE students.

## OBJECTIVE

The objective of this article is to systematically review the evidence available regarding the impact of PA-related interventions to improve mental health and QoL outcomes in HE students to determine the following research questions:

1. Are PA or exercise interventions effective in improving the mental health and QoL of HE students?
2. Does effectiveness of the interventions vary according to the length and type of the intervention?

## METHODS

This systematic review was based on a published protocol in PROSPERO—International prospective register of systematic reviews (registration number: CRD42022325975) following PRISMA guidelines (Page *et al*., 2021).

### Data sources

An exhaustive search was conducted within six databases: ProQuest, MEDLINE, Embase, CINAHL, SPORTDiscus and CENTRAL, only including peer-reviewed journal articles published up until May 2022. The search strategy was developed through a combination of keywords for each database using the Boolean operators ‘OR’ and ‘AND’: (University students OR College students) AND (Physical activity OR exercise OR movement OR physical fitness) AND (Health-related quality of life OR Stress OR depression OR anxiety) AND (Intervention OR Programme OR randomised controlled trial (RCT) OR non-randomised controlled trial (non-RCT)). Only manuscripts written in English were considered. Two reviewers independently assessed articles for initial study inclusion based on title and abstract. Full texts were then retrieved and assessed based on their eligibility for inclusion.

### Study inclusion and exclusion criteria

#### Type of participants

Any study including on-campus or remote learning HE students (≥18 years old) full-time or part-time, and undergraduate or postgraduate students were included. Students studying vocational training courses or short courses (i.e. less than 6 months duration) were excluded.

#### Type of interventions

Interventions deemed eligible for inclusion had to include a PA or movement-based component, aiming to improve student’s mental health and/or QoL. Interventions of all lengths and mode of delivery (e.g. online, on-campus, etc.) were included.

#### Type of studies

Original quantitative studies including RCTs and non-RCTs were eligible for inclusion. Cross-sectional studies, systematic reviews and meta-analyses were not included.

#### Type of outcome

This review focuses on the psychological effects of the intervention received relating to the mental health and/or QoL of HE students.

### Data extraction

The search results were exported to Zotero® to eliminate duplicates. Titles were screened and eligible studies were downloaded onto excel, reviewed for any remaining duplicates missed by Zotero (S.D.) and the abstracts were manually screened by two researchers independently (S.D. and M.K.). If there was any discrepancy, a third investigator (K.P.) was called to reach a mutual consensus amongst the research team. The full texts of these articles were retrieved. Subsequently, the reference lists of selected studies were reviewed (S.D.) to identify additional relevant studies. From all the eligible full-texts, data were extracted by three researchers (S.D., M.K., K.P.). The following summary data were considered: country, study design, sample size, gender, age (range and mean (standard deviation: SD)), diagnoses, intervention characteristics, analysis, outcomes and effectiveness of intervention.

### Data analysis

Across all study designs the purpose of the study, specific population of interest (within HE students); type, duration, and follow-up period of the intervention; and specific mental health measures are described with consideration to key attributes of robustness and generalisability. The reported effectiveness of interventions is described for RCT and non-RCT studies, with the comparators also of interest for the RCTs. The theoretical frameworks underpinning interventions are also described. Due to the heterogeneity of study designs, interventions and outcome measures a meta-analysis was not possible.

### Risk of bias (ROB)

ROB was assessed, whereby three reviewers (S.D., M.K., K.P.) reviewed all included studies. Thereafter, the reviewers discussed the assessment of the included studies to come to a final agreement of the assessment of each paper. ROB for RCTs was assessed using the revised version 2 of the Cochrane ROB for randomized trials (RoB 2) (Sterne *et al*., 2019). The ROB of all included non-RCTs was assessed using the ROBINS-I tool (Sterne *et al*., 2016). This tool provides a systematic way to organize and present the available evidence relating to ROB, and by signalling questions, answers can help identify areas of concern regarding ROB. Similarly, to the RoB 2 tool, outcomes for each individual domain are generated alongside an overall ROB outcome.

## RESULTS

### Results of literature search

**Fig. 1:**
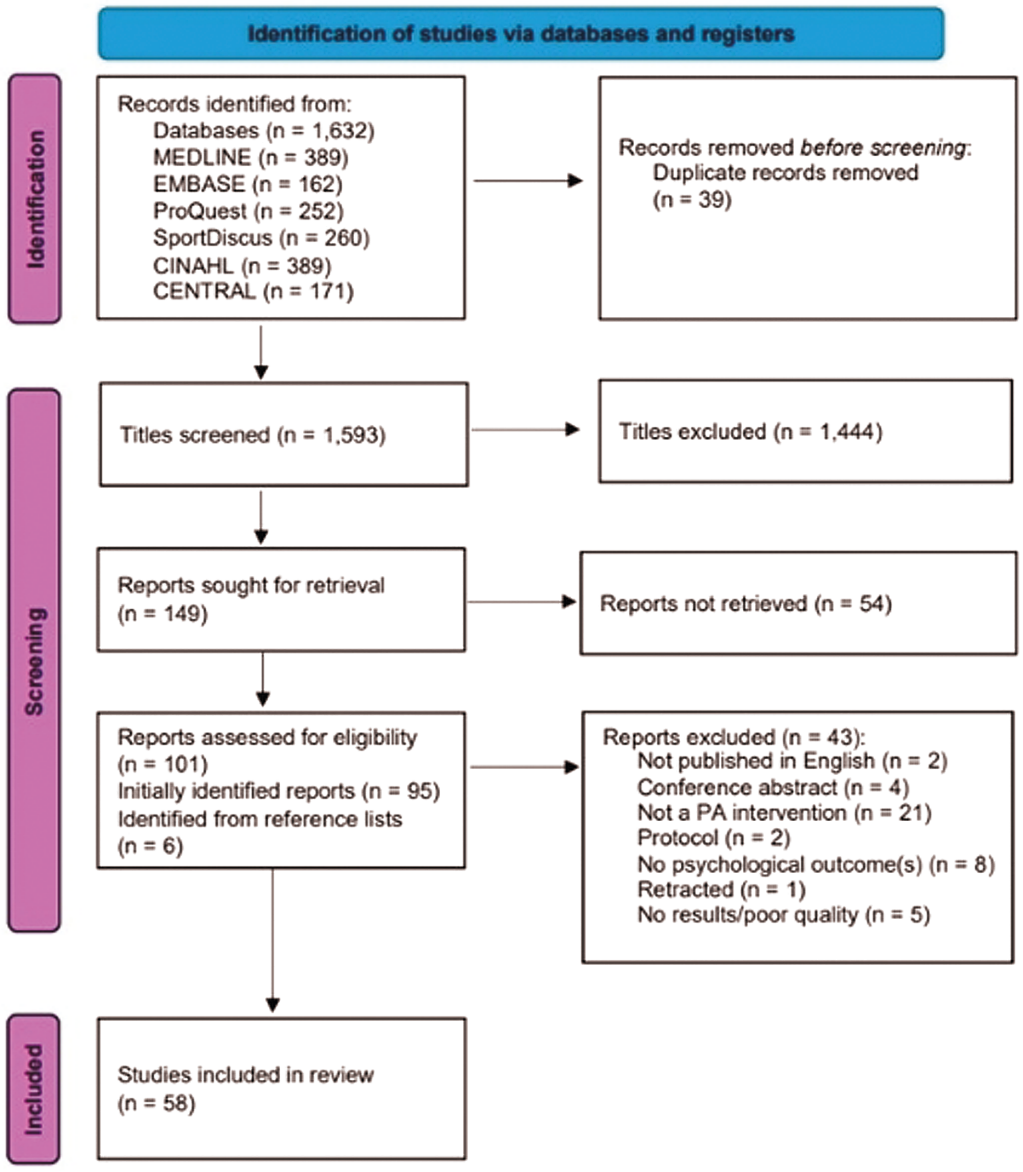
PRISMA flow diagram.

The total search retrieved 1,632 records. Following the removal of duplicates, 1,593 records were screened by title, of which 1,444 titles were excluded, and the remaining 149 abstracts were retrieved and screened. The remaining 101 full-text articles were screened, of which 58 publications from 1991 to 2023 met the inclusion criteria (see Figure 1). These publications included 38 RCT studies (Crocker and Grozelle, 1991; Brown *et al*., 1993; Kim *et al*., 2004; Mailey *et al*., 2010; Akandere and Demir, 2011; Hemat-Far *et al*., 2012; Kim *et al*., 2013; Gallego *et al*., 2014; Zheng *et al*., 2015; Li *et al*., 2015; de Vries *et al*., 2016, 2018; Sharp and Caperchione, 2016; von Haaren *et al*., 2016; Huang *et al*., 2017; López-Rodríguez *et al*., 2017; Albracht-Schulte and Robert-McComb, 2018; Schmalzl *et al*., 2018; Dinani *et al*., 2019; Eather *et al*., 2019; Faro *et al*., 2019; Herbert *et al*., 2020; Wan Yunus *et al*., 2020; Zimmermann and Mangelsdorf, 2020; Zheng and Ji, 2021; Fukui *et al*., 2021; Saltan and Ankaralı, 2021; Xiao *et al*., 2021; Ji *et al*., 2022) and 20 non-RCT studies (O’Connor *et al*., 1995; Bass *et al*., 2002; Wang *et al*., 2004; Caldwell *et al*., 2009; Tayama *et al*., 2012; Koschel *et al*., 2017; Ezati *et al*., 2020; Muir *et al*., 2020; deJonge *et al*., 2021; Marschin and Herbert, 2021; Martínez-Díaz and Carrasco, 2021; Salehian *et al*., 2021; Tong *et al*., 2021; Forseth *et al*., 2022; La Count *et al*., 2022).

### Characteristics of included studies

Characteristics of the included studies are summarized in Table 1. Of the 58 included studies, most (*n* = 38, 66%) were RCTs. Furthermore, most studies (*n* = 17) were from the USA followed by 11 in China, and 6 in Canada. Four studies were from Iran, and three studies were included from both Germany and Spain. Two studies came from Japan, Turkey and the Netherlands, and the remaining countries were associated with one study each; Australia, Belgium, India, Korea, Malaysia, Norway, Taiwan and the UK (see Table 1). Only one RCT study reported the use of behaviour change theory (Mailey *et al*., 2010), which was social cognitive theory. No non-RCT studies employed behaviour change theory. Intervention duration ranged from 1 single 10-min session to 20 weeks of intervention. Seven of the RCT (Kim *et al*., 2013; Mailey *et al*., 2010; Philippot *et al*., 2022; Sun *et al*., 2023; Wan Yunus *et al*., 2020; Zhang *et al.*, 2023a; Zhu *et al*., 2023) and 8 of the non-RCT (Wang *et al*., 2004; deJonge *et al*., 2021; Martínez-Díaz and Carrasco, 2021; Forseth *et al*., 2022; Danielsen *et al*., 2023; Gurung *et al*., 2023; Sandra *et al*., 2023; Strehli *et al*., 2023) studies were designed as pilot trials.

**Table 1:** Summary details of included studies

Study ID	Country	Design	Participants analysed; median group size^1^	Female %	Age mean (SD)^2^	Inclusion criteria^3^	Exclusion criteria^3^	Incentive	Intervention	Comparators	Session length (min) × sessions per week × weeks: total dose (min)^4^	Effective^5^
RCT												
Akandere and Demir (2011)	Turkey	–	120; 60	50	Ns; 20–24	Active (conservatory students)	–	–	Dance	Usual routine	110 × 3 × 12: 3,960	Yes
Albracht (2018)	The USA	Crossover	40; 40	100	20.2 (1.97)	–	Medical (phys & psych)	–	Yoga	Quiet rest	30 × 1 × 1: 30	No
Brown *et al.* (1993)	The USA	Crossover	10; 10	50	24.9 (5.8)	Previously active but injury/illness	–	–	Running or cycling	Quiet rest	~20 × 1 × 1: 20	No
Chawla *et al.* (2022)	India	–	30; 15	70	24.1 (1.2)	–	Medical (phys & psych)	–	Squat exercises with whole body vibration	Squat exercises	NSL × 2 × 4: ~240	No
Crocker and Grozelle (1991)	Canada	–	85; 28	52	20.9 (NS)	–	–	–	Aerobic	Relaxation; control (do anything for 30 min)	30–40 × 1 × 1: 35	Yes
de Vries *et al.* (2018)	The Netherlands	Waitlist control	99; 49.5	81	20.9 (2.30)	Novice, fatigue (high) (not treated)	Medical (phys)	–	Running	Usual routine	1 × 3 × 6: 18	Yes
de Vries *et al.* (2016)	The Netherlands	Waitlist control	97; 48	81	20.1 (2.35)	Fatigue (high)	Active, medical (phys)	–	Running	Usual routine	60 × 3 × 6: 1,080	Yes
Dinani *et al.* (2019)	Iran	–	64; 32	NS	21.5 (NS)	Novice	Medical (phys & psych)	–	Tai Chi	Usual routine	40 × 3 × 8: 960	Yes
Eather *et al.* (2019)	Australia	Waitlist control	53; 26.5	66	20.4 (1.88)	.		–	HIIT	Usual routine	8–12 × 3 × 8: 240	No
Faro *et al.* (2019)	The USA	Crossover	34; 34	100	27 (4.5)		Active	-	Functional resistance training	Traditional resistance training	32 × 1 × 1: 32	Yes
Fukui *et al.* (2021)	Japan	–	125; 62.5	54	21.6 (2.9)	–	–	–	Home workout	Usual routine	27.7 (average) × variable 0–7 × 8: 222	Yes
Gallego *et al.* (2014)	Spain	–	125; 42	58	20.1 (3.68)	–	–	Credit	Sports games	Mindfulness; usual routine	60 × 1 × 8: 480	Yes
Hemat-Far *et al.* (2012)	Iran	–	20; 10	100	Ns; 18–45	Major depression, novice	–	–	Running	Usual routine	40–60 × 3 × 8: 1,200	Yes
Herbert *et al.* (2020)	Germany	Concurrent online & lab pilot studies	104; 20	95	22.6 (3.68)	Novice	Medical (phys & psych)	Prize	Aerobics	Expression writing; motor coordination	8–12 × 2 × 6: 120	Yes
Huang *et al.* (2017)	Taiwan	–	337; 167.5	Ns	Ns; >20	Novice	Medical (phys & psych)	$	Exergames	Usual routine	30 × 1 × 2: 60	Yes
Ji *et al.* (2022)	China	–	84; 14	39	24.2 (2.53)	Anxiety (not medicated)	Injury, medical (phys)	–	Circuit training	6 combinations of intensity and frequency	60 × 1 × 6: 360	Yes
Kim *et al.* (2013)	The USA	Pilot, waitlist control	18; 9	Ns	24.8 (9.41)	Anxiety	–	$	Kouk Sun Do	Usual routine	70 × 2–3 × 4: 700	Yes
Kim *et al.* (2004)	Korea	–	54; 27	100	Ns	–	Medical (phys & psych)	–	Meridian exercise	Usual routine	30 × 2 × 6: 360	No
Li *et al.* (2021)	China	–	387; 193.5	49	23.5 (3.5)	–	–	–	Baduanjin	Health knowledge course	45 × 5 × 12: 2,700	Yes
Li *et al.* (2015)	China	–	206; 103	83	20.8 (1.10)	–	Active	–	Baduanjin	Usual routine	60 × 5 × 12: 3,600	No
Lopez- Rodriguez *et al.* (2017)	Spain	Waitlist control	95; 47.5	75	22.3 (4.12)	Distress	Medical (phys & psych)	–	Dance	Usual routine	90 × 1 × 4: 360	Yes
Mailey *et al.* (2010)	The USA	Pilot study	47; 23.5	68	25 (Ns)	Current mental health counselling	–	$	Pedometer	Mental health counselling	NSL × NSL × 10: -	No
Mota *et al.* (2023)	Canada	Block randomization by sex	80; 40	86	18.1 (1.46)	–	Medical (phys & psych)	$, membership	Mobile health app	Usual routine	NSL × NSL × 12: -	No
Murray *et al.* (2022)	The USA	–	74; 37	85	23.5 (6.0)	–	–	–	Aerobic	Yoga mindfulness	30 × 2 × 8: 480	No
Philippot *et al.* (2022)	Belgium	Pilot	28; 14	89	20.8 (1.7)	Anxiety	–	–	HIIT	Usual routine	10 × 3 × 4: 120	Yes
Saltan and Ankarali (2021)	Turkey	–	92; 35	82	19.0 (1.76)	Novice	Medical (phys)	–	Pilates; therapeutic exercises	Usual routine	Unclear × 3 × 12: 720	Yes
Schmalz *et al.* (2018)	The USA	Stratified random assignment (age/sex/handedness)	40; 19	57	24.3 (5.72)	Novice	Medical (ADHD)	–	Yoga	Ujjayi breath	45 × 2 × 8: 720	No
Sharp and Caperchio (2016)	Canada	–	137; 68.5	53	18 (0.69)	–	–	Prize	Pedometer	Usual routine	NSL × NSL × 12: -	No
Sun *et al.* (2023)	China	–	37; 18.5	51	20.9 (4.3)	Sedentary, severe anxiety	Medical (phys)	–	Qigong	Usual routine	60 × 5 × 12: 3,600	Yes
von Harren (2016)	Germany	–	61; 30	0	21.4 (1.8)	–	–	Credit	Aerobic	Usual routine	30 × 2 × 20: 1,200	Yes
Wan Yunus *et al.* (2020)	Malaysia	Pilot	36; 18	86	22.9 (1.05)	Not overweight or obese	Medical (phys & psych)	–	Exergames	Usual routine	30 × 3 × 6: 540	Yes
Xiao *et al.* (2021)	China	–	96; 33	26	19.3 (1.29)	Novice	Medical (phys & psych)	–	Baduanjin; basketball	Usual routine	90 × 3 × 12: 3,240	Yes
Zhang *et al.* (2023a)	China	–	73; 36.5	100	19.2 (18–20)	Novice	Medical (phys)	–	Baduanjin	Usual routine	60 × 3 × 12: 2,160	Yes
Zhang *et al.* (2023b)	China	Pilot	18; 9	72	23.3 (5.1)	Anxiety or depression	Medical (phys & psych)	–	Tai Chi	Usual routine	60 × 5 × 8: 2,400	No
Zhao *et al.* (2022)	China	Waitlist control	86; 29	71	21.2 (2.1)	Sedentary, mild depression	Medical (phys & psych)	–	Aerobic; resistance	Usual routine	40–60 × 3 × 12: 1,800	Yes
Zheng *et al.* (2015)	China	–	198; 97.5	67	20.6 (1.1)	Novice	Medical (phys)	–	Tai Chi	Usual routine	60 × 5 × 12: 3,600	No
Zhu *et al.* (2023)	The USA	Pilot	27; 13.5	89	21.9 (2.42)	Elevated (but not clinical) anxiety or depression	Medical (phys)	$	Online guided HIIT	Self-guided HIIT	50 × 3 × 8: 1,200	No
Zimmerman and Mangelsdorf (2020)	The USA	–	60; 30	73	20.1 (1.48)	Included mental health disorders		Credit	Creative movement	Art	20 × 1 × 1: 20	No
Non-RCT												
Bass *et al.* (2002)	The USA	Unclear allocation, implied self-selection	114; 35	60	Ns	Self-enrolled into weight training subject	–	–	Weight training, dance	Usual routine	45 × 3 × 8: 1,080	Yes
Caldwell *et al.* (2009)	The USA	Self-enrolled into classes used as groups	98; 29	51	21.3 (2.24)	Self-enrolled into PA subject	–	–	Pilates, Tai Chi	Usual routine	50 × 2–3 × 15: 1,875	Yes (Pilates)
Danielsen *et al.* (2023)	Norway	Pilot, single group	10; 10	70	24.5 (5.1)	Seeking mental health support	Medical (phys)	–	Aerobic and strength	No comparator	60 × 2 × 10: 1,200	Yes
de Jonge *et al.* (2021)	Canada	Pilot, single group	68; 68	82	23.0 (3.42)	Seeking mental health support	Active	–	Aerobic or resistance	No comparator	30 × 1 × 6: 180	Yes
Ezati *et al.* (2020)	Iran	Block allocation (two dormitories)	67; 33.5	100	20.3 (1.46)	Not obese	Medical (phys & psych)	–	Aerobic	Usual routine	60 × 3 × 4: 720	Yes
Forseth *et al.* (2022)	The USA	Pilot, single group	12; 12	71	23.8 (4.6)	–	Medical (phys & psych)	–	Yoga	No comparator	60 × 2 × 8: 960	Yes (pre-post)
Gao *et al.* (2022)	China	Self-selected groups	89; 44.5	100	19.8 (1.13)	Female	Medical (phys & psych)	–	Aromatherapy yoga	Yoga	90 × 1 × 12: 1,080	No
Gurung *et al.* (2023)	The UK	Pilot, small groups	15; 7.5	79	Ns	–	Medical (phys & psych)	–	Running	No comparator	65 × 1 × 10: 650	Yes (pre-post)
Koschel *et al.* (2017)	The USA	Self-selected groups	15; 7.5	NS	28.3 (6.56)	–	–	–	Choice	Usual routine	5–50 × 3 × 1: 75	No
La Count *et al.* (2022)	The USA	ADHD participants age & sex matched to non-ADHD controls	36; 18	50	20.8 (1.70)	ADHD or non-ADHD	Medical (medication)	–	HIIT	Comparison between AHDH/non-ADHD	19 × 1 × 1: 19	No
Marschin and Herbert (2021)	Germany	Self-enrolled into classes used as groups	20; 10	85	23.0 (1.65)	–	Medical (phys)	Prize, credit	Home’ workout	Expressive writing	5–10 × 1 × 9: 68	No
Martinez-Diaz and Carrasco (2021)	Spain	Pilot, single group	25; 25	0	21.7 (2.1)	Active	Medical (phys & psych), taking exams	–	HIIT	No comparator	10 × 1 × 1: 10	No
Muir *et al.* (2020)	Canada	Single group	49; 49	65	23.1 (4.97)	Seeking mental health support (low risk), novice	–	–	Circuit/gym	No comparator	45–60 × 3 × 6: 945	Yes
O’connor *et al.* (1995)	The USA	Single group	32; 16	59	NS	Novice to Expert (3 experiments)	–	–	HIIT (maximal training)	No comparator	40 × 3 × 8: 960	No
Salehian *et al.* (2021)	Iran	RCT of participants with high coronavirus anxiety	45; 15	100	NS	Coronavirus anxiety	Medical (phys & psych)	-	Tai Chi	cognitive-spiritual; usual routine	30–40 × 2 × 5: 350	Yes
Sandra *et al.* (2023)	Canada	Pilot, single group	49; 49	80	23.1 (4.0)	Low PA	Medical (phys & psych)	$	Aerobic and resistance training	No comparator	45–50 × 3 × 2: 270	Yes (pre-post)
Strehli *et al.* (2023)	The USA	Pilot	21; 21	81	21.0 (2.20)	–	–	$	Yoga and qigong	No comparator	10 × 3 × 8: 240	Yes (pre-post)
Tayama *et al.* (2012)	Japan	Single group	39; 39	100	20.3 (0.7)	–	–	–	Pedometer	No comparator	NSL × NSL × 1: -	No
Tong *et al.*, (2021)	China	Self-enrolled into classes used as groups	334; 85	71	20.0 (1.40)	Novice	Medical (phys & psych)	–	Yoga	Aerobic	60 × 1 × 1–12: 720	Yes
Wang *et al.* (2004)	The USA	Pilot, single group	30; 30	63	24.2 (2.74)	–	Medical (phys)	-	Tai Chi	No comparator	60 × 2 × 12: 1,440	Yes (pre-post)

^1^Number of participants in the analysis and the median group size as there are slight variations between groups in most studies.

^2^Mean age and standard deviations were pooled across groups statistics if overall demographics were not given; NS = not stated; age range (min–max) is stated in lieu of other summary statistics where available.

^3^Novice is used to indicate the participants did not need any experience or regular activity; Expert is used to indicate that participants had significant experience in a relevant activity; Medical (phys & psych) indicates that there were medical exclusions for physical and psychological indications, these varied between studies.

^4^Intervention dose is given as the minutes per activity session, number of sessions per week and number of weeks of the intervention. This was used to estimate an approximate total length of intervention in minutes. Note these numbers are indicative only as some study designs had variable length sessions and sessions per week; or increased duration/intensity over the length of the study. Abbreviation NSL = no set length.

^5^Indicated if the study found a statistically significant difference on one or more psychological outcomes between comparator groups; if there was no comparator group then this indicates effective pre–post comparison as indicated. Psychological measurements with estimated effect sizes for effective RCT studies are listed on a separate table.

^6^Description of study indicates it was a randomized controlled trial, however authors describe as quasi-experimental. Due to ambiguity, it is listed with the non-RCT studies.

#### Incentive to participate

Only 13 (22%) studies outlined small incentives to participate which included the award of additional course credits, money, prize draws (e.g. amazon voucher) and free yoga classes (see Table 1).

#### Diagnoses, psychological outcomes and measurements

Eighteen studies (31%) required participants to have a psychological diagnosis or experience including being referred by the on-campus mental health team, experiencing anxiety, experiencing moderate depression, or having attention deficit hyperactivity disorder (ADHD). Other studies had inclusion criteria based on activity levels or previous exercise experience (*n* = 16, 28%) and the remainder (*n* = 24, 41%) had no specific inclusion criteria other than being students, although recruitment may have been restricted to sub-groups of students (such as course enrolment or dormitories). Most of the studies (*n* = 48, 83%) reported psychological outcomes as their primary outcome, including perceived stress, depression, state anxiety and QoL. In other studies (*n* = 10, 17%), non-psychological measures such as cardiorespiratory fitness, heart rate variability and aerobic capacity were reported as their primary outcome, with psychological outcomes being secondary outcomes. Various psychological measurements were used (see Table 2) including the Spielberger State-Trait Anxiety Inventory (STAI) and Beck Depression Inventory (BDI).

**Table 2:** Summary of psychological instruments used and the language adaptation if known, if no language is stated it is assumed to be in English based on study location

Study ID	Psychological outcomes are primary outcomes	State Trait Anxiety Inventory (STAI)	Beck Depression Inventory (BDI)	Perceived Stress Scale (PSS)	Depression Anxiety Stress Scale (DASS)	Other
**RCTs**						
Akandere and Demir (2011)	Yes		BDI (Turkish)			
AlbrachtSchulte and Robert-McComb (2018)	Yes	STAI-Y1				
Brown *et al*. (1993)	Yes	STAI				
Chawla *et al*. (2022)	Yes				DASS-42	SF-36
Crocker and Grozelle (1991)	Yes	STAI				
de Vries *et al*. (2018)	Yes					Six single-item indicators of well‐being covering fatigue, energy, stress, health status, satisfaction and self‐efficacy on Dutch Grade Notation-Based Scale.
de Vries *et al*. (2016)	Yes					General Self-efficacy (GSE) (Dutch) Utrecht Burnout Scale modified for students (UBOS-S) (Dutch) Fatigue Assessment Scale (FAS-10) Need for Recovery Scale (6 items) Adapted Sleep Quality Scale (6 items)
Dinani *et al*. (2019)	Yes				DASS-42 (Persian)	
Eather *et al*. (2019)	No	STAI		PSS-14		
Faro *et al*. (2019)	Yes	STAI-Y1				Feeling Scale (FS-11)
Fukui *et al*. (2021)	No					SF-8 (Japanese) WHO-5 (Japanese) Kessler Screening Scale for Psychological Distress (K6)
Gallego *et al*. (2014)	Yes				DASS-21 (Spanish)	
Hemat-Far *et al*. (2012)	Yes		BDI			
Herbert *et al*. (2020)	Yes	STAI (German)	BDI-II (German)			The Positive and Negative Affect Schedule (PANAS) (German) Quality of Life (WHOQOL-BREF) (German) Stress and Coping Inventory (SCI) (German)
Huang *et al*. (2017)	Yes			Items from PSS (Taiwanese)		Scale of Shacham (vigour) (Taiwanese) Items from Lyubomirsky and Lepper (happiness) (Taiwanese)
Ji *et al*. (2022)	Yes					Beck Anxiety Inventory (BAI) Self-rating Depression Scale (SDS) Pittsburgh Sleep Quality Index (PSQI) (Language unclear)
Kim *et al*. (2013)	Yes					State of Anxiety (SAI) Depression Status Inventory (DSI) Self-esteem Inventory (SEI) (Language unclear)
Kim *et al*. (2004)	Yes	STAI	BDI-II			General Self-efficacy (GSE)
Li *et al*. (2021)	Yes					Coronavirus Anxiety Scale (CAS) Psychological Well-being Scale (PWBS)
Li *et al*. (2015)	Yes			CPSS (Chinese)		General Self-efficacy (GSE) (Chinese) Profile of Mood States (POMS)(Chinese) Quality of Life (WHOQOL-BREF) (Chinese) The Symptom Checklist-90 (SCL-90) (Chinese) Schulte Grid (8*8) test (attention) Self-Esteem Scale (SES) Pittsburgh Sleep Quality Index (PSQI) (Chinese)
Lopez-Rodriguez *et al*. (2017)	Yes			PSS (European Spanish)		Centre for Epidemiologic Studies Depression Scale (CES-D) (Spanish) Pittsburgh Sleep Quality Index (PSQI) (Spanish)
Mailey *et al*. (2010)	Yes	STAI	BDI			Exercise Self-Efficacy Scale Barriers Self-Efficacy Scale
Mota *et al*. (2023)	Yes					Stress Indicator Questionnaire (SQI)
Murray *et al*. (2022)	Yes					Generalized Anxiety Disorder Scale (GAD-7) Major Depression Inventory (MDI)
Philippot *et al*. (2022)	No				DASS-21	Screened using Generalized Anxiety Disorder Scale (GAD-7)
Saltan and Ankarali (2021)	No		BDI (Turkish)			Nottingham Health Profile (Turkish)
Schmalzl *et al*. (2018)	Yes			PSS-14		
Sharp and Caperchione (2016)	Yes					GHQ-12
Sun *et al*. (2023)	Yes					SF-36 Hamilton Anxiety Rating Scale (HAM-A) Fatigue Scale 14 (FS-14) Pittsburgh Sleep Quality Index (PSQI)
von Haaren *et al*. (2016)	No					Electronic diaries with stress ratings scales Language unclear
Wan Yunus *et al*. (2020)	Yes				DASS-21 (Malay)	Functional Outcome Sleep Questionnaire (FOSQ) Language unclear
Xiao *et al*. (2021)	Yes			CPSS-14 (Chinese)		Self-Rating Anxiety Scale UCLA Loneliness Scale adapted Feelings of Inadequacy Scale (FIS)
Zhang *et al*. (2023a)	No					Symptom Checklist-90 (SCL90)
Zhang *et al*. (2023b)	Yes					Zung’s Self-rating Anxiety Scale (SAS) Zung’s Self-rating Depression Scale (SDS)
Zhao *et al*. (2022)	Yes					Zung Self-Rating Depression Scale (SDS) Neuroticism Extraversion Openness Five Factor Inventory (NEO-FFI) International Physical Activity Questionnaire-Short Form (IPAQ-SF)
Zheng *et al*. (2015)	Yes					General Self Efficacy (GSE)(Chinese) Quality of Life (WHOQOL-BREF) (Chinese) Self-Esteem Scale (SES)
Zhu *et al*. (2023)	Yes					Counseling Center Assessment of Psychological Symptoms (CCAPS-34)
Zimmerman and Mangelsdorf (2020)	Yes					Perceived Intensity of the Stressor PANAS
**Non-RCTs**						
Bass *et al*. (2002)	Yes					The Survey of Recent Life Experiences
Caldwell *et al*. (2009)	Yes					Four-item self-regulatory efficacy instrument Self-efficacy measurements specific to either Pilates or Taiji Quan were developed by the authors (these are not on comparable scales) Four-Dimensional Mood Scale
Danielsen *et al*. (2023)	No					Hopkins Symptoms Checklist-25 (HSCL-25) Warwick-Edinburgh Well-being Scale (WEMWBS) Satisfaction with Life Scale (SWLS)
deJonge *et al*. (2021)	Yes					The Mental Health Inventory (MHI-38)
Ezati *et al*. (2020)	Yes					Pittsburgh Sleep Quality Index (PSQI) (Persian) Multidimensional Fatigue Inventory (MFI-20) (Persian)
Forseth *et al*. (2022)	Yes		BDI-II	PSS-14		
Gao *et al*. (2022)	Yes			PSS-14		Pittsburgh Sleep Quality Index (PSQI)
Gurung *et al*. (2023)	Yes					Generalized Anxiety Disorder Scale-7 (GAD-7) Patient Health Questionnaire-9 (PHQ-9)
Koschel *et al*. (2017)	Yes			PSS-14		
La Count *et al*. (2022)	Yes				DASS-21 (modified)	
Marschin and Herbert (2021)	Yes		BDI-II (German)	PSS (German)		Quality of Life (WHOQOL-BREF) (German) Stress and Coping Inventory (SCI) (German) The Eating Disorder Inventory-2 (EDI-2) (German) PANAS (German)
Martinez-Diaz and Carrasco (2021)	No					POMS (Language unclear)
Muir *et al*. (2020)	Yes					Mental Health Inventory-38 (MHI-38)
O’Connor (1995)	Yes	STAI				
Salehian *et al*. (2021)	Yes					General Health Questionnaire (GHQ-28) (Persian) Corona Disease Anxiety Scale (CDAS8) (Persian)
Sandra *et al*. (2023)	Yes		BDI-II			Hospital Anxiety and Depression Scale (HADS)
Strehli *et al*. (2023)	No			PSS-4		WHO Well-Being Index (WHO-5)
Tayama *et al*. (2012)	No					General Self-efficacy (GSE) (Japanese) Psychological Stress Response Scale (SRS-18) (Japanese)
Tong *et al*. (2021)	Yes					Stress (adapted scale) Mindful Attention Awareness Scale Self-Compassion Scale Emotions (adapted scale) Language unclear
Wang *et al*. (2004)	Yes					SF-36

#### Intervention effectiveness

Interventions were effective in 36 studies (62%) for improving at least one measure of mental health or QOL in HE students (Akandere and Demir, 2011; Bass *et al*., 2002; Caldwell *et al*., 2009; Crocker and Grozelle, 1991; Danielsen *et al*., 2023; de Vries *et al*., 2016, 2018; deJonge *et al*., 2021; Dinani *et al*., 2019; Ezati *et al*., 2020; Faro *et al*., 2019; Forseth *et al*., 2022; Fukui *et al*., 2021; Gallego *et al*., 2014; Gurung *et al*., 2023; Hemat-Far *et al*., 2012; Herbert *et al*., 2020; Huang *et al*., 2017; Ji *et al*., 2022; Kim *et al*., 2013; Li *et al*., 2022; López-Rodríguez *et al*., 2017; Muir *et al*., 2020; Philippot *et al*., 2022; Salehian *et al*., 2021; Saltan and Ankaralı, 2021; Sandra *et al*., 2023; Strehli *et al*., 2023; Sun *et al*., 2023; Tong *et al*., 2021; von Haaren *et al*., 2016; Wan Yunus *et al*., 2020; Wang *et al*., 2004; Xiao *et al*., 2021; Zhang and Jiang, 2023b; Zhao *et al*., 2022). Five of these studies did not have a comparator so the effectiveness relates only to changes over time (pre-post) and not between groups. Where an intervention was found to be effective the effect sizes were typically medium to large, in either an adjusted repeated measures analysis or a less robust sequence of paired *t*-tests. No studies which used sequences of *t*-tests adjusted for multiple comparisons. The effect sizes as reported by the authors or estimated from available information are outlined in (Supplementary File S1: SF1). Nearly half the studies (48%) compared a PA intervention with the ‘usual routine’ (including waitlist control groups). The remaining studies used a variety of comparators including between different PA interventions (e.g. basketball with Taichi) and non-PA interventions such as art, therapy and expressive writing (Table 1). For the purposes of this review, we have categorized these interventions based on the intervention and not the comparator. These are: (a) moderate-vigorous intensity PA (MVIPA) intervention, (b) high-intensity interval training (HIIT), (c) mind–body PA interventions and (d) miscellaneous interventions.

##### MVIPA interventions.

There were 29 studies which used MVIPA interventions including aerobic strength and resistance exercises, Pilates, running, dance, circuit/gym training and sports games. Of these studies, 25 were found to be effective (Crocker and Grozelle, 1991; Bass *et al*., 2002; Caldwell *et al*., 2009; Akandere and Demir, 2011; Hemat-Far *et al*., 2012; Gallego *et al*., 2014; de Vries *et al*., 2016, 2018; von Haaren *et al*., 2016; Huang *et al*., 2017; López-Rodríguez *et al*., 2017; Faro *et al*., 2019; Ezati *et al*., 2020; Herbert *et al*., 2020; Muir *et al*., 2020; Wan Yunus *et al*., 2020; deJonge *et al*., 2021; Fukui *et al*., 2021; Saltan and Ankaralı, 2021; Xiao *et al*., 2021; Ji *et al*., 2022; Zhao *et al*., 2022; Danielsen *et al*., 2023; Gurung *et al*., 2023; Sandra *et al*., 2023). Four studies did not find significant intervention effects. Two of these studies compared an aerobic intervention with a yoga intervention (Tong *et al*., 2021; Murray *et al*., 2022); 1 study compared a home workout with expressive writing (Marschin and Herbert, 2021); and 1 study used cycling/running interventions (Brown *et al*., 1993). Notably the cycling/running intervention did not include general student populations, rather it recruited physically challenged students, enrolled in an adaptive physical education class for a single 20-min session.

Nineteen of the 29 studies in this group were RCTs (Crocker and Grozelle, 1991; Brown *et al*., 1993; Akandere and Demir, 2011; Hemat-Far *et al*., 2012; Gallego *et al*., 2014; de Vries *et al*., 2016, 2018; von Haaren *et al*., 2016; Huang *et al*., 2017; López-Rodríguez *et al*., 2017; Faro *et al*., 2019; Herbert *et al*., 2020; Wan Yunus *et al*., 2020; Fukui *et al*., 2021; Saltan and Ankaralı, 2021; Xiao *et al*., 2021; Ji *et al*., 2022; Murray *et al*., 2022, 2022; Zhao *et al*., 2022) and 17 were found to be effective (Crocker and Grozelle, 1991; Akandere and Demir, 2011; Hemat-Far *et al*., 2012; Gallego *et al*., 2014; de Vries *et al*., 2016, 2018; von Haaren *et al*., 2016; Huang *et al*., 2017; López-Rodríguez *et al*., 2017; Faro *et al*., 2019; Herbert *et al*., 2020; Wan Yunus *et al*., 2020; Fukui *et al*., 2021; Saltan and Ankaralı, 2021; Xiao *et al*., 2021; Ji *et al*., 2022; Zhao *et al*., 2022). One was explicitly a pilot study (Wan Yunus *et al*., 2020). There were 10 non-RCTs (Bass *et al*., 2002; Caldwell *et al*., 2009; Ezati *et al*., 2020; Muir *et al*., 2020; deJonge *et al*., 2021; Marschin and Herbert, 2021; Tong *et al*., 2021; Danielsen *et al*., 2023; Gurung *et al*., 2023; Sandra *et al*., 2023), 4 of which were pilots (deJonge *et al*., 2021; Danielsen *et al*., 2023; Gurung *et al*., 2023; Sandra *et al*., 2023), 8 were found to be effective (Bass *et al*., 2002; Caldwell *et al*., 2009; Ezati *et al*., 2020; Muir *et al*., 2020; deJonge *et al*., 2021; Danielsen *et al*., 2023; Gurung *et al*., 2023; Sandra *et al*., 2023), and 2 did not show statistically significant improvements (Marschin and Herbert, 2021; Tong *et al*., 2021).

##### High-intensity interventions.

HIIT interventions (*n* = 6) included three RCTs (Eather *et al*., 2019; Philippot *et al*., 2022; Zhu *et al*., 2023), two of which were pilot (Philippot *et al*., 2022; Zhu *et al*., 2023) and three non-RCTs (Martínez-Díaz and Carrasco, 2021; La Count *et al*., 2022), one of which was a pilot (Martínez-Díaz and Carrasco, 2021). Only the RCT pilot intervention (Philippot *et al*., 2022) showed effectiveness for HIIT interventions. The oldest study in this group describes a procedure of sequential maximal exercises similar to HIIT, although published prior to the popularization of the term (O’Connor *et al*., 1995). They found that this training *increased* anxiety immediately post-training for low-fitness students, although anxiety returned to baseline at follow-up. This study also included maximal treadmill exercises with both highly trained runners and physically fit students who also did not find the sessions effective in reducing anxiety.

##### Mind–body PA interventions.

There were 19 studies which used mind–body interventions (Albracht-Schulte and Robert-McComb, 2018; Caldwell *et al*., 2009; Dinani *et al*., 2019; Forseth *et al*., 2022; Gao *et al*., 2022; Kim *et al*., 2013; Kim *et al*., 2004; Li *et al*., 2015, 2022; Salehian *et al*., 2021; Schmalzl *et al*., 2018; Strehli *et al*., 2023; Sun *et al*., 2023; Tong *et al*., 2021; Wang *et al*., 2004; Xiao and Zheng, 2022; Zhang *et al.*, 2023a; Zheng *et al*., 2015; Zhang and Jiang, 2023) including various forms of yoga, Tai-Chi, Qigong (including Baduanjin), Kouk Sun Do, and meridian exercise, where studies (Caldwell *et al*., 2009; Tong *et al*., 2021; Xiao *et al*., 2021) were also included in the previous section as they were comparisons between mind–body interventions and MVIPA interventions. Overall, these studies provide mixed evidence of effectiveness with only 6 out of 12 RCTs finding at least one significant improvement in a mental health or QOL measure against the comparator (see Supplementary File S1: SF1). Eight studies compared with the usual routine, and one each for quiet rest, health education or ujjayi breath. Most of these interventions were lengthy with typically 60-min sessions 2–5 times per week for 8–12 weeks. There were seven non-RCTs, with five claiming the intervention was effective, however, three of these were pilot studies with no comparator. Tong and colleagues (2021) found yoga to be superior to aerobic-style exercises however students self-selected into groups and the effect was primarily considered to be through increased mindfulness rather than a direct measure of mental health or QOL.

Two studies which did not find Tai Chi or Baduanjin to be effective were from the same research group and rigorously designed with similar published protocols (Zheng *et al*., 2013, 2014). They included long interventions (each 12 weeks), the largest sample sizes (approximately 200 participants each) and intention to treat analyses. In both cases, the authors posit that differences with the control group may have been difficult to detect as there were no limits on what activities the control group may have been involved with outside of the trial. The background of the participants may be an important consideration as they were students of traditional Chinese medicine, whereas the three trials which were found to be effective involved nursing (Kim *et al*., 2004; Dinani *et al*., 2019) or general university students (Xiao *et al*., 2021). These studies had smaller sample sizes (approximately 30 students per group) and ran for 6, 8 and 12 weeks, respectively. There was also a pilot trial with a small sample size of 18 students which found evidence for the effectiveness of Kouk Sun Do in improving the mental health of students (Kim *et al*., 2013). The non-RCT studies which found some evidence for the effectiveness of Tai Chi included a pilot trial with no comparator (Wang *et al*., 2004) and, an Iranian study looking specifically at Corona-disease anxiety which did not find Tai Chi effective in comparison with cognitive-spiritual therapy (Salehian *et al*., 2021) but was more effective than the control (receiving no intervention). Overall, the evidence is mixed and suggests that mind–body exercises may be effective for improving students’ mental health, however, this may depend on the background of students and which activities they already usually participate in.

##### Miscellaneous interventions.

There were seven miscellaneous studies including five RCTs (Mailey *et al*., 2010; Sharp and Caperchione, 2016; Zimmermann and Mangelsdorf, 2020; Chawla *et al*., 2022; Mota *et al*., 2023) and two non-RCTs (Tayama *et al*., 2012; Koschel *et al*., 2017). None of these studies found the interventions to significantly improve HE students’ mental health and/or QoL. There were three pedometer-based interventions (Mailey *et al*., 2010; Tayama *et al*., 2012; Sharp and Caperchione, 2016) which did not specify a number of steps or intervention lengths to participants. It could be argued these are not truly movement-based interventions, but rather tracking-based interventions (with the aim that tracking might increase movement). Similarly, Mota and colleagues (2023) trialled a mobile health app as an intervention which included exercise videos but did not specify the amount of exercise to complete. Another two studies were considered in this category as they involved only a single session or event. One compared a single 20-min creative movement versus art (Zimmermann and Mangelsdorf, 2020) and whilst both groups improved over time there was no difference between the groups. The other allowed students to choose activities within a 3-day on-campus event and whilst it is not called a pilot or feasibility trial, the sample size was 15 students (Koschel *et al*., 2017). The final study compared squat exercises with and without whole-body vibration and both groups improved in the domains of depression, anxiety and stress (Chawla *et al*., 2022). In this case, the intervention is the addition of the whole-body vibration in conjunction with the exercises, which was not effective.

### Assessment of ROB

ROB assessment for included RCT studies is summarized (see Supplementary File S2: SF2) and outlined in relation to each RCT study (see Supplementary File S3: SF3). Most of the RCT studies included (*n* = 22; 58%) scored low in overall ROB, with a further 10 having an unclear ROB, and 6 having high ROB. It is important however to consider that it not possible to blind participants to these types of interventions. Assessment of ROB for included non-RCT studies is summarized (see Supplementary File S4: SF4) and outlined in relation to each non-RCT study (see Supplementary File S5: SF5). Similarly to the RCT studies, overall, most of the non-RCTs reported low ROB (*n* = 12; 60%), whereas five were unclear and the remaining three had a high ROB.

## DISCUSSION

This systematic review suggests that exercise interventions, which are MVIPA, can positively impact the mental health and/or QoL of HE students. Interventions include Pilates, aerobic exercises, basketball, weight, resistance and gym training, dance, exercise games and home workouts and running. Mixed results were observed for mind–body interventions in improving the mental health and/or QoL of HE students. However, there was substantial variability between studies in relation to the context, sample size, intervention duration and outcomes. Interventions involving HIIT were not found to be effective, except for a single pilot study. However, there were only three studies (Eather *et al*., 2019; Philippot *et al*., 2022; Zhu *et al*., 2023) trialling HIIT in a similar design to other movement-based interventions (in terms of duration) so this area needs further research. Interventions which gave participants access to tracking their physical activity (such as pedometers or mobile app) which did not have a specific session length or duration were not effective (Mailey *et al*., 2010; Tayama *et al*., 2012; Sharp and Caperchione, 2016; Mota *et al*., 2023). Overall, there is substantial variety in the type, duration and measurement of PA interventions, but there is evidence that these can improve aspects of mental health with an appropriate program.

## STRENGTHS AND LIMITATIONS OF PRIMARY RESEARCH

PA trials rely upon the participants volunteering to participate and in turn, conclusions about effectiveness cannot be drawn for students in general as they may not have the internal motivation to initiate or maintain participation in such activities. One study (Ezati *et al*., 2020) used a block allocation of students based on dormitories and still found the intervention effective. Further studies of this type could offer insights into general adherence and attrition; however, it may be more challenging in settings where students are not ‘captive’ based on residence. This is discussed further in recommendations. Additionally, most studies fail to detail information surrounding participants’ regular activities and the activities of the control groups after not being assigned to the intervention arm.

Many of the RCT studies included in this review use validated instruments to measure psychological outcomes (Table 2), including the STAI, PSS and BDI. Of the studies included, many included interventions administered over several weeks (≥20 weeks). This is an individual strength within studies only, as such a wide array of instruments are used making any comparisons between studies problematic. Additionally, many studies used multiple measures and sequences of paired *t*-tests, inflating the type 1 error rate (i.e. false positives). Assessment of multiple psychological outcome measures also raises questions surrounding the accuracy of the results as participants may experience survey fatigue.

## STRENGTH AND LIMITATIONS OF THIS SYSTEMATIC REVIEW

A strength of this systematic review is the inclusion of exhaustive searches for relevant peer-reviewed journal articles in six library databases. Although searches were restricted to articles written in the English language only, research findings from a range of different countries where English is not the first language are included. The majority of included studies (*n* = 34 studies, 58.6%) had low ROB. Only nine studies (15.5%) were found to have high ROB.

This review has several limitations. First, it was not feasible to conduct meta-analyses due to heterogeneity in study designs, interventions, outcome measures and types of analysis conducted. Second, variations in the length and intensity of interventions limits direct comparison across all included studies. Lastly, this systematic review includes only peer-reviewed journal articles, so publication bias may be a potential limitation of the findings since studies with negative or inconclusive findings may be less likely to have been published (DeVito and Goldacre, 2019).

## RECOMMENDATIONS FOR FUTURE PRACTICE

This review supports the ongoing evaluation of MVIPA interventions for the mental health and wellbeing of HE students. There is evidence that these can be effective for students who are interested in participating. Given the numerous forms of PA, it is valuable to have ongoing research on different interventions. However, it would be helpful to use standardized instruments (e.g. STAI, BDI, PSS and DASS) so that future meta-analysis is possible, and to continue to run trials which continue for several weeks. There is currently only limited evidence of the effectiveness of PA as a general health-promotion activity aimed at all students for mental health. Further research is needed on feasibility, acceptability and adherence within this framework. One study used a block allocation of dormitories (Ezati *et al*., 2020) and the intervention group did have slightly higher non-compliance than the control group (i.e. 5 vs 1 students, respectively) but the overall sample size is still relatively low (i.e. 67 students). Additionally, two studies used entire classes as experimental groups (Marschin and Herbert, 2021; Tong *et al*., 2021), however, these were fitness and psychology classes, respectively, and may not be directly applicable to students in non-health-related fields. From a health-promotion lens, it would be valuable to understand which activities would encourage participation and adherence, and if students who are experiencing symptoms of stress, anxiety and depression can be encouraged to participate.

Most studies found a gender bias in participation (i.e. greater proportion of females than males). This limitation is not unusual. A systematic review on the prevalence of mental health problems in undergraduate students found that more than half had greater than 60% female participation (Sheldon *et al*., 2021). The only intervention in this review which skewed to a greater proportion of males (without being exclusively male) included basketball as an intervention (Xiao *et al*., 2021). A systematic review of PA interventions for physical health also found that more than half of the studies included predominantly female participants (Plotnikoff *et al*., 2015), and that overall interest in the interventions was relatively low. It is possible that a PA intervention explicitly aimed at improving mental health rather than physical health may attract wider and more diverse participation. It would also be of interest to hear student perspectives on the ‘attractiveness’ of programs which are promoted for reducing stress and anxiety, rather than reducing weight. Loneliness was only considered explicitly in one of the studies in this review (Xiao *et al*., 2021), however, this is also a growing concern for HE students in wider research (Bernardon *et al*., 2011; Diehl *et al*., 2018).

Pragmatic trials which report initial interest, engagement throughout the semester and the student perspective would complement the existing research on efficacy. It is unlikely that a single PA intervention would be appealing to all students, however, a range of activities which could include a social element, may influence uptake and regular attendance. In this review, only one study offered a range of activities (Koschel *et al*., 2017). Although it did not find that the program was effective in reducing stress on quantitative measures before exams, it was only a 3-day intervention with a small sample size and the qualitative feedback found that all students in the intervention group felt it had reduced stress. This approach may provide a framework for a larger and longer program.

## CONCLUSION

This systematic review offers the first detailed synthesis on PA and exercise-specific interventions targeted at improving the mental health and wellbeing of HE students. The evidence suggests that these interventions can positively impact the mental health and QoL of HE students. Evidence shows those interventions which include MVIPA to be the most effective, including aerobics, dance, basketball and running. Mind–body exercises including yoga, Tai Chi and Qigong may also be effective depending on context however this evidence is mixed. HIIT and pedometer/tracking interventions were not effective in improving psychological outcomes in HE students. Implemented long-term and widely across HE institutions, MVIPA interventions may improve mental health in HE students.

## Supplementary Material

daae027_suppl_Supplementary_Files_1

daae027_suppl_Supplementary_Files_2

daae027_suppl_Supplementary_Files_3

daae027_suppl_Supplementary_Files_4

daae027_suppl_Supplementary_Files_5

daae027_suppl_Supplementary_Files_6

daae027_suppl_Supplementary_Files_7

## Data Availability

Data will be made available upon request from the authors.
