# Supplementary material for: The effectiveness of physical activity interventions in improving higher education students’ mental health: A systematic review
Source: Health Promot Int. 2024 Apr 2;39(2):daae027. doi: 10.1093/heapro/daae027 (PMC10985680; doi:10.1093/heapro/daae027)
Supplement: daae027_suppl_Supplementary_Files_1 [file daae027_suppl_supplementary_files_1.docx]

**Table 3.** Summary of RCTs effectiveness

| Study ID | Psychological outcomes are primary outcomes | Effect size as reported by the original study from a multivariable analysis (partial eta squared) | Estimated Cohen's d (between groups at post) |
| --- | --- | --- | --- |
| Akandere & Demir (2011)^48^ | yes | no adjusted analysis | depression: 0.53 (medium) dance vs usual routine |
| Crocker & Grozelle (1991)^45^ | yes | adjusted analysis presented without effect sizes | anxiety: 0.31 (small) aerobic vs relaxation anxiety: 1.35 (large) aerobic vs usual routine |
| de Vries et al., (2018)^54^ | yes | adjusted analysis presented without effect sizes | fatigue: 0.86 (large) running vs usual routine health status: 0.74 (large) running vs usual routine satisfaction: 0.47 (medium) running vs usual routine energy: 0.67 (large) running vs usual routine stress: 0.25 (small) running vs usual routine self-efficacy: 0.12 (small) running vs usual routine |
| de Vries et al., (2016)^39^ | yes | multivariable analysis  3 out 7 measures significant: overall fatigue: 0.5 (medium) running vs usual routine need for recovery: 0.9 (medium) running vs usual routine sleep quality: 0.9 (medium) running vs usual routine |  |
| Dinani et al., (2019)^50^ | yes | multivariable analysis presented without effect sizes | depression: 0.59 (medium) tai chi vs usual routine anxiety: 0.65 (large) tai chi vs usual routine stress: 0.7 (large) tai chi vs usual routine self-confidence: 1.08 (large) tai chi vs usual routine |
| Faro et al., (2019)^40^ | yes | no multivariable analysis | affect: 0.02 (small) functional vs traditional resistance training anxiety: 0.01 (small) functional vs traditional resistance training |
| Fukui et al., (2021)^28^ | no | multivariable analysis presented without effect sizes;  3 out of 14 measures significant | general health: 0.17 (small) home workout vs usual routine wellbeing: 0.17 (small) home workout vs usual routine distress: 0.29 (small) home workout vs usual routine |
| Galego et al., (2014)^31^ | yes | multivariable analysis presented without direct effect sizes of interest; 1 of the 3 sub-measures significant | stress: 0.61 (medium) sports games vs mindfulness stress: 0.04 (small) sports games vs usual routine |
| Hemat-Far et al., (2012)^36^ | yes | no multivariable analysis | depression: 1.04 (large) running vs usual routine |
| Herbert et al., (2020)^46^ | Yes | Multivariable analyses presented with varying small to large effect sizes from 0.03 to 0.13 across experiments and measures |  |
| Huang et al., (2017)^41^ | yes | multivariable analysis presented without effect sizes; 2 out of 3 measures significant | insufficient details to estimate effect sizes |
| Ji et al., (2022)^44^ | yes | multivariable analysis presented with large effect sizes ranging from 0.35 to 0.85 indicating dose-response relationship for exercise intensity in improving anxiety, depression and sleep quality; and exercise frequency improving anxiety and depression |  |
| Kim et al., (2013)^26^ | yes | multivariable analysis presented with large effect sizes ranging from 0.33 to 0.44 for anxiety and depression with no significant effect on general self-efficacy |  |
| Li et al., (2021)^92^ | yes | no multivariable analysis; 3 of 3 measures significant | wellbeing: 2.31 (large) Baduanjin vs usual routine coronavirus anxiety: 0.19 (small) Baduanjin vs usual routine pain: 3.24 (large) Baduanjin vs usual routine |
| Lopez-Rodriguez et al., (2017)^34^ | yes | no multivariable analysis; 2 out of 3 measures significant | depression: 0.56 (medium) dance vs usual routine anxiety: 0.44 (medium) dance vs usual routine |
| Philippot et al., (2022)^81^ | no | no multivariable analysis; 1 of 3 measures significant | stress: 0.34 (small) HIIT vs usual routine |
| Saltan & Ankarali (2021)^30^ | no | no multivariable analysis | depression: 1.08 (large) Pilates vs usual routine depression: 0.64 (medium) therapeutic exercises vs usual routine general health: 1.01 (large) Pilates vs usual routine general health: 0.6 (medium) therapeutic exercises vs usual routine |
| Sun et al., (2023)^75^ | yes | no multivariable analysis; 3 out of 6 measures significant | anxiety: 0.73 (large) qigong vs usual routine sleep quality: 0.72 (large) qigong vs usual routine bodily pain: 0.9 (large) qigong vs usual routine |
| Von Haaren et al., (2016)^29^ | no | psychological measure used as an independent variable in estimating effect of exercise on physiological stress response |  |
| Wan Yunus et al., (2020)^43^ | yes | multivariable analysis with Cohen's d calculated between groups | depression: 0.63 (medium) exergames vs usual routine anxiety: 1.09 (large) exergames vs usual routine stress: 0.72 (medium) exergames vs usual routine sleep: 0.87 (large) exergames vs usual routine |
| Xiao et al., (2021)^33^ | yes | multivariable analysis presented without effect sizes | 0.67-2.27 (large) each intervention vs usual routine across all measures 0.59-0.79 (medium) basketball vs Baduanjin across all measures |
| Zhang & Jiang et al., (2023a)^77^ | no | no multivariable analysis; 6 out of 9 measures significant | somatization: 0.4 (medium) Baduanjin vs usual routine obsessive-compulsive: 0.57 (medium) Baduanjin vs usual routine interpersonal sensitivity: 0.14 (small) Baduanjin vs usual routine depression: 0.93 (large) Baduanjin vs usual routine anxiety: 0.36 (medium) Baduanjin vs usual routine phobic anxiety: 0.69 (large) Baduanjin vs usual routine |
| Zhao et al., (2022)^78^ | yes | multivariable analysis presented with large effect sizes from 0.26-0.52 for depression for interventions compared with control (but not between interventions) |  |
