# Supplementary figures and images for: The effectiveness of physical activity interventions in improving higher education students’ mental health: A systematic review
Source: Health Promot Int. 2024 Apr 2;39(2):daae027. doi: 10.1093/heapro/daae027 (PMC10985680; doi:10.1093/heapro/daae027)

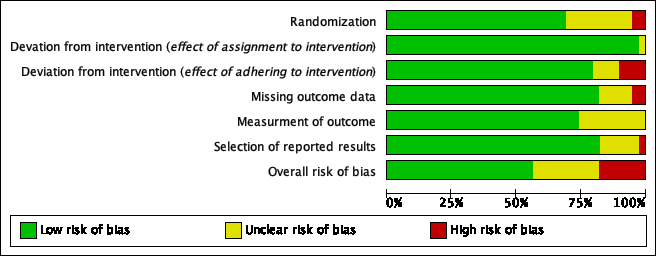


**Supplementary File 1**. RCT risk of bias graph

Supplement: daae027_suppl_Supplementary_Files_2 [file daae027_suppl_supplementary_files_2.docx]

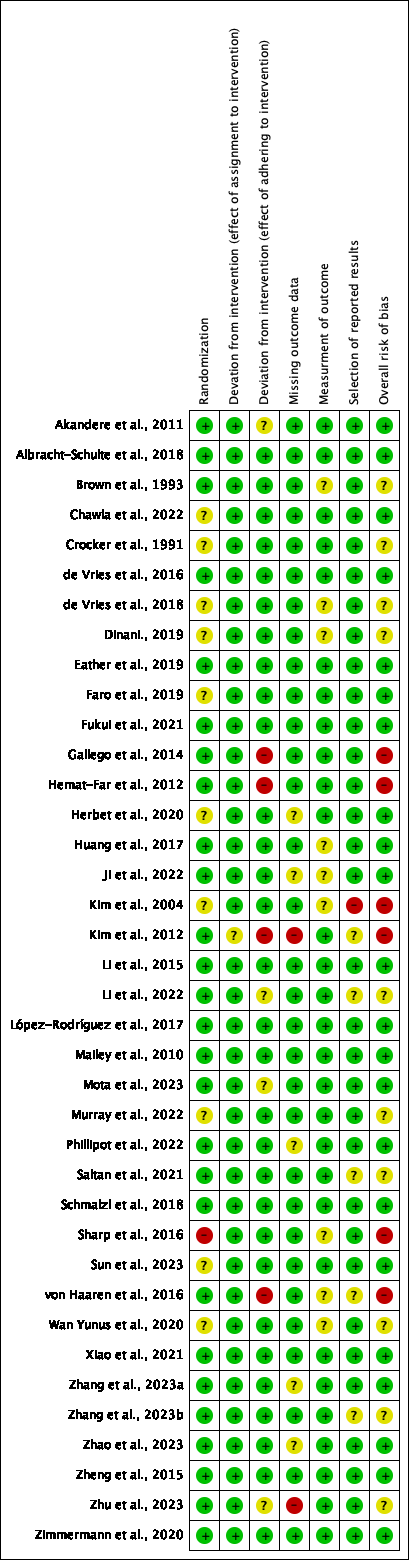


**Figure 2.** RCT ROB

Supplement: daae027_suppl_Supplementary_Files_3 [file daae027_suppl_supplementary_files_3.docx]

**
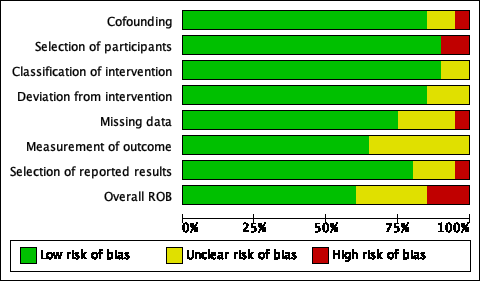
**

**Supplementary File 2**. Non-RCT risk of bias graph

Supplement: daae027_suppl_Supplementary_Files_4 [file daae027_suppl_supplementary_files_4.docx]

**
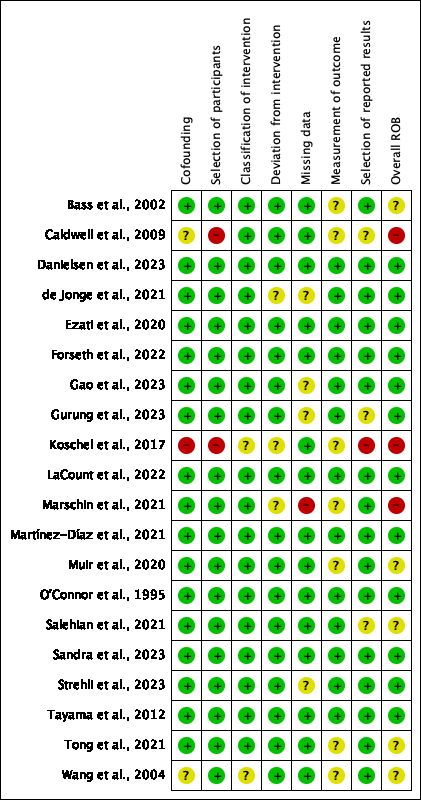
**

**Figure 3.** Non-RCT ROB

Supplement: daae027_suppl_Supplementary_Files_5 [file daae027_suppl_supplementary_files_5.docx]
