# Supplementary material for: The effectiveness of physical activity interventions in improving higher education students’ mental health: A systematic review
Source: Health Promot Int. 2024 Apr 2;39(2):daae027. doi: 10.1093/heapro/daae027 (PMC10985680; doi:10.1093/heapro/daae027)
Supplement: daae027_suppl_Supplementary_Files_6 [file daae027_suppl_supplementary_files_6.docx]

**Supporting Information**

Search History for ProQuest

| # | Searches | Results | Type |
| --- | --- | --- | --- |
| 1 | ((university students) or (college students)) | 81,664 | Advanced |
| 2 | ((physical activity) or (physical fitness) or (exercise) or (movement)) | 2,415,722 | Advanced |
| 3 | ((health-related quality of life) or (stress) or (depression) or (anxiety)) | 1,945,113 | Advanced |
| 4 | ((intervention) or (prorgamme) or (program) or (randomi?ed controlled trial) or (non-randomi?ed controlled trial)) | 3,091,841 | Advanced |
| 1 and 2 and 3 and 4 | summary((university students) OR (college students)) AND summary((physical activity) OR (physical fitness) OR (exercise) OR (movement)) AND summary((health-related quality of life) OR (stress) OR (depression) OR (anxiety)) AND summary((intervention) OR (programme) OR (program) OR (randomi?ed controlled trial) OR (non-randomi?ed controlled trial)) | 259 | Advanced |
